# (*tert*-Butyl­imido)bis­(η^5^-cyclo­penta­dien­yl)pyridine­zirconium(IV)

**DOI:** 10.1107/S1600536810033556

**Published:** 2010-08-28

**Authors:** Katharina Kaleta, Perdita Arndt, Anke Spannenberg, Uwe Rosenthal

**Affiliations:** a Leibniz-Institut für Katalyse e. V. an der Universität Rostock, Albert-Einstein-Strasse 29a, 18059 Rostock, Germany

## Abstract

The title compound, [Zr(C_5_H_5_)_2_(C_4_H_9_N)(C_5_H_5_N)], was obtained from the reaction of (C_5_H_5_)_2_Zr(py)(η^2^-Me_3_SiC_2_SiMe_3_) (py is pyridine) and ^*t*^BuN=C=N^*t*^Bu alongside the formation of (C_5_H_5_)_2_Zr(CN^*t*^Bu)(η^2^-Me_3_SiC_2_SiMe_3_). The zirconium atom is coordinated in a distorted tetra­hedral geometry by two cyclo­penta­dienyl ligands, a pyridine ligand, and a *tert*-butyl­imido ligand *via* a Zr=N double bond. The *tert*-butyl group is disordered over two positions in a 0.634 (5):0.366 (5) ratio.

## Related literature

For other metallocene complexes (C_5_H_5_)Cp*M*(*L*)(N^*t*^Bu) (Cp = C_5_H_5_, C_5_Me_5_; *M* = Ti, *L* = py; *M* = Zr, *L* = py, thf (thf is tetrahydrofuran), *exo*-norbornene oxide) with an *M*=N double bond, see: Blum *et al.* (2003 ▶, 2005 ▶); Dunn *et al.* (1997 ▶); Krska *et al.* (1998 ▶); Walsh *et al.* (1988 ▶, 1993 ▶); Zuckerman *et al.* (2000 ▶). For the structure of (*rac*-ebthi)Zr(py)(N^*t*^Bu) (ebthi = ethyl­enebis(η^5^-tetra­hydro­inden­yl)), see: Hoyt *et al.* (2004 ▶). For the preparation of the starting material (C_5_H_5_)_2_Zr(py)(η^2^-Me_3_SiC_2_SiMe_3_), see: Rosenthal *et al.* (1995 ▶). For the characterization of the by-product  (C_5_H_5_)_2_Zr(CN^*t*^Bu)(η^2^-Me_3_SiC_2_SiMe_3_) of the above-described reaction, see: Bach *et al.* (2007 ▶).
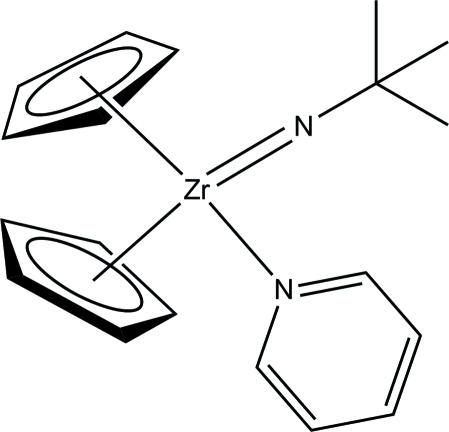


## Experimental

### 

#### Crystal data

[Zr(C_5_H_5_)_2_(C_4_H_9_N)(C_5_H_5_N)]
*M* *_r_* = 371.62
Orthorhombic, 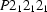

*a* = 9.3946 (2) Å
*b* = 13.6156 (4) Å
*c* = 14.4126 (3) Å
*V* = 1843.56 (8) Å^3^
*Z* = 4
Mo *K*α radiation
μ = 0.60 mm^−1^
*T* = 200 K
0.50 × 0.50 × 0.35 mm

#### Data collection

Stoe IPDS II diffractometer
Absorption correction: numerical (*X-SHAPE* and *X-RED32*; Stoe & Cie, 2005 ▶) *T*_min_ = 0.730, *T*_max_ = 0.896
35444 measured reflections
4990 independent reflections
4752 reflections with *I* > 2σ(*I*)
*R*_int_ = 0.025

#### Refinement

*R*[*F* ^2^ > 2σ(*F* ^2^)] = 0.023
*wR*(*F* ^2^) = 0.059
*S* = 1.03
4990 reflections
181 parameters
16 restraints
H-atom parameters constrained
Δρ_max_ = 0.45 e Å^−3^
Δρ_min_ = −0.38 e Å^−3^
Absolute structure: Flack (1983 ▶), 216 Friedel pairs
Flack parameter: −0.03 (4)

### 

Data collection: *X-AREA* (Stoe & Cie, 2005 ▶); cell refinement: *X-AREA*; data reduction: *X-AREA*; program(s) used to solve structure: *SHELXS97* (Sheldrick, 2008 ▶); program(s) used to refine structure: *SHELXL97* (Sheldrick, 2008 ▶); molecular graphics: *XP* in *SHELXTL* (Sheldrick, 2008 ▶); software used to prepare material for publication: *SHELXTL*.

## Supplementary Material

Crystal structure: contains datablocks I, global. DOI: 10.1107/S1600536810033556/im2220sup1.cif

Structure factors: contains datablocks I. DOI: 10.1107/S1600536810033556/im2220Isup2.hkl

Additional supplementary materials:  crystallographic information; 3D view; checkCIF report

## Figures and Tables

**Fig. 1.**
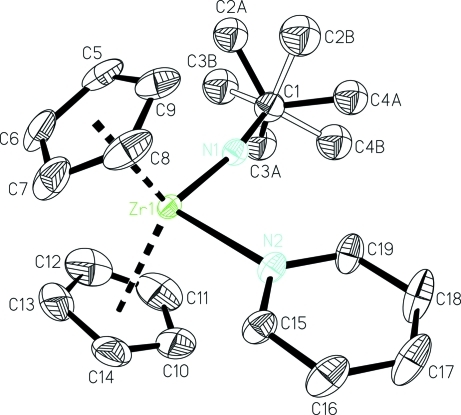
Molecular structure of the title compound showing the atom-labelling scheme. Hydrogen atoms are omitted for clarity. Thermal ellipsoids are drawn at the 30% probability level.

## supplementary crystallographic information

### Comment

We studied the reaction of several carbodiimides with metallocene precursors as (C_5_H_5_)_2_M(η^2^-Me_3_SiC_2_SiMe_3_) (M = Ti, Zr) to synthesize and characterize new metallacycles with heteroatoms. In this case the reaction revealed a C—N bond cleavage which resulted in two products. Additionally to (C_5_H_5_)_2_Zr(py)(N*^t^*Bu) the complex (C_5_H_5_)_2_Zr(CN*^t^*Bu)(η^2^-Me_3_SiC_2_SiMe_3_) was found which was described by Bach *et al.* (2007).

The title compound consists of a zirconium center coordinated by two cyclopentadienyl ligands, a stabilizing pyridine and a *tert*-butyl imido ligand. The geometry at the zirconium atom is distorted tetrahedral (main deviations from the expected value of 109.47° are obtained in N1—Zr1—N2 95.64 (6)° and Cp—Zr1—Cp 123.9°). Bond lengths and angles can be compared to the thf stabilized complex (C_5_H_5_)_2_Zr(thf)(N*^t^*Bu) described by Walsh *et al.* (1993). The bond lengths Zr1—N1 with 1.843 (2) Å and N1—C1 with 1.434 (3) Å are not significantly different compared to those of (C_5_H_5_)_2_Zr(thf)(N*^t^*Bu) (Zr—N 1.826 (4) and N—C 1.449 (6) Å). In the title compound the Zr1—N1—C1 angle of 168.93 (13)° is about 5° smaller than the corresponding angle found for the almost linear *tert*-butyl imido ligand in (C_5_H_5_)_2_Zr(thf)(N*^t^*Bu).

### Experimental

To a solution of 235 mg (0.5 mmol) (C_5_H_5_)_2_Zr(py)(η^2^-Me_3_SiC_2_SiMe_3_) in 10 ml of *n*-hexane was added dropwise 0.1 ml (0.5 mmol) of *^t^*BuN=C=N*^t^*Bu. The reaction mixture was allowed to stand for 16 h. During this period the solution turned red and yellow crystals were formed which were isolated, washed with cold *n*-hexane and dried in vacuo. Yield: 46% (85 mg, 0.229 mmol).

### Refinement

All H atoms were placed in idealized positions with d(C—H) = 0.98 (CH_3_) and 0.95 Å (CH) and refined using a riding model with *U*_iso_(H) fixed at 1.5 *U*_eq_(C) for CH_3_ and 1.2 *U*_eq_(C) for CH.

### Crystal data

**Table:**

[Zr(C_5_H_5_)_2_(C_4_H_9_N)(C_5_H_5_N)]	*F*(000) = 768
*M**_r_* = 371.62	*D*_x_ = 1.339 Mg m^−^^3^
Orthorhombic, *P*2_1_2_1_2_1_	Mo *K*α radiation, λ = 0.71073 Å
Hall symbol: P 2ac 2ab	Cell parameters from 11751 reflections
*a* = 9.3946 (2) Å	θ = 2.0–29.6°
*b* = 13.6156 (4) Å	µ = 0.60 mm^−^^1^
*c* = 14.4126 (3) Å	*T* = 200 K
*V* = 1843.56 (8) Å^3^	Prism, yellow
*Z* = 4	0.50 × 0.50 × 0.35 mm

### Data collection

**Table:**

Stoe IPDS II diffractometer	4990 independent reflections
Radiation source: fine-focus sealed tube	4752 reflections with *I* > 2σ(*I*)
graphite	*R*_int_ = 0.025
ω scans	θ_max_ = 29.2°, θ_min_ = 2.1°
Absorption correction: numerical (*X-SHAPE* and *X-RED32*; Stoe & Cie, 2005)	*h* = −12→12
*T*_min_ = 0.730, *T*_max_ = 0.896	*k* = −18→18
35444 measured reflections	*l* = −19→19

### Refinement

**Table:**

Refinement on *F*^2^	Secondary atom site location: difference Fourier map
Least-squares matrix: full	Hydrogen site location: inferred from neighbouring sites
*R*[*F*^2^ > 2σ(*F*^2^)] = 0.023	H-atom parameters constrained
*wR*(*F*^2^) = 0.059	*w* = 1/[σ^2^(*F*_o_^2^) + (0.043*P*)^2^ + 0.044*P*] where *P* = (*F*_o_^2^ + 2*F*_c_^2^)/3
*S* = 1.03	(Δ/σ)_max_ = 0.001
4990 reflections	Δρ_max_ = 0.45 e Å^−^^3^
181 parameters	Δρ_min_ = −0.38 e Å^−^^3^
16 restraints	Absolute structure: Flack (1983), 2169 Friedel pairs
Primary atom site location: structure-invariant direct methods	Flack parameter: −0.03 (4)

### Special details

**Table:**

Geometry. All e.s.d.'s (except the e.s.d. in the dihedral angle between two l.s. planes) are estimated using the full covariance matrix. The cell e.s.d.'s are taken into account individually in the estimation of e.s.d.'s in distances, angles and torsion angles; correlations between e.s.d.'s in cell parameters are only used when they are defined by crystal symmetry. An approximate (isotropic) treatment of cell e.s.d.'s is used for estimating e.s.d.'s involving l.s. planes.
Refinement. Refinement of *F*^2^ against ALL reflections. The weighted *R*-factor *wR* and goodness of fit *S* are based on *F*^2^, conventional *R*-factors *R* are based on *F*, with *F* set to zero for negative *F*^2^. The threshold expression of *F*^2^ > σ(*F*^2^) is used only for calculating *R*-factors(gt) *etc*. and is not relevant to the choice of reflections for refinement. *R*-factors based on *F*^2^ are statistically about twice as large as those based on *F*, and *R*- factors based on ALL data will be even larger.

### Fractional atomic coordinates and isotropic or equivalent isotropic displacement parameters (Å^2^)

**Table:**

	*x*	*y*	*z*	*U*_iso_*/*U*_eq_	Occ. (<1)
C2A	−0.0298 (4)	0.5132 (4)	0.2054 (4)	0.0710 (13)*	0.634 (5)
H2A1	−0.0198	0.5691	0.2477	0.107*	0.634 (5)
H2A2	−0.1118	0.4734	0.2241	0.107*	0.634 (5)
H2A3	−0.0441	0.5373	0.1420	0.107*	0.634 (5)
C3A	0.1191 (6)	0.4163 (4)	0.3089 (2)	0.0765 (14)*	0.634 (5)
H3A1	0.1243	0.4733	0.3502	0.115*	0.634 (5)
H3A2	0.2062	0.3772	0.3152	0.115*	0.634 (5)
H3A3	0.0366	0.3760	0.3256	0.115*	0.634 (5)
C4A	0.0964 (5)	0.3633 (3)	0.1445 (3)	0.0690 (13)*	0.634 (5)
H4A1	0.1846	0.3253	0.1493	0.103*	0.634 (5)
H4A2	0.0841	0.3862	0.0806	0.103*	0.634 (5)
H4A3	0.0155	0.3219	0.1620	0.103*	0.634 (5)
C2B	0.0023 (10)	0.4490 (9)	0.1272 (6)	0.095 (3)*	0.366 (5)
H2B1	0.0528	0.4262	0.0717	0.143*	0.366 (5)
H2B2	−0.0348	0.5153	0.1162	0.143*	0.366 (5)
H2B3	−0.0769	0.4044	0.1408	0.143*	0.366 (5)
C3B	0.0215 (10)	0.4865 (7)	0.2929 (5)	0.081 (3)*	0.366 (5)
H3B1	−0.0046	0.5556	0.2843	0.122*	0.366 (5)
H3B2	0.0806	0.4799	0.3486	0.122*	0.366 (5)
H3B3	−0.0650	0.4470	0.3001	0.122*	0.366 (5)
C4B	0.1428 (12)	0.3436 (3)	0.2234 (6)	0.078 (2)*	0.366 (5)
H4B1	0.1974	0.3200	0.1700	0.117*	0.366 (5)
H4B2	0.0556	0.3047	0.2297	0.117*	0.366 (5)
H4B3	0.2003	0.3370	0.2798	0.117*	0.366 (5)
C1	0.1041 (2)	0.45092 (16)	0.20903 (15)	0.0500 (5)	
C15	0.6237 (2)	0.54813 (15)	0.02869 (15)	0.0469 (4)	
H15	0.6509	0.6142	0.0403	0.056*	
C16	0.7109 (3)	0.49094 (19)	−0.02490 (19)	0.0627 (7)	
H16	0.7964	0.5172	−0.0498	0.075*	
C17	0.6736 (3)	0.39523 (18)	−0.04233 (19)	0.0737 (9)	
H17	0.7332	0.3538	−0.0784	0.088*	
C18	0.5476 (3)	0.36108 (19)	−0.00621 (19)	0.0722 (9)	
H18	0.5181	0.2955	−0.0181	0.087*	
C19	0.4643 (3)	0.42177 (15)	0.04710 (15)	0.0493 (5)	
H19	0.3776	0.3970	0.0717	0.059*	
N1	0.22557 (16)	0.51038 (11)	0.18718 (11)	0.0361 (3)	
N2	0.50127 (18)	0.51501 (12)	0.06570 (11)	0.0383 (3)	
Zr1	0.363638 (15)	0.605190 (11)	0.172085 (11)	0.03033 (5)	
C5	0.16807 (18)	0.72069 (14)	0.12010 (16)	0.0645 (8)	
H5	0.0771	0.7076	0.1462	0.077*	
C6	0.2709 (2)	0.78328 (13)	0.15691 (17)	0.0720 (9)	
H6	0.2625	0.8204	0.2125	0.086*	
C7	0.3884 (2)	0.78188 (13)	0.09794 (17)	0.0748 (9)	
H7	0.4741	0.8179	0.1062	0.090*	
C8	0.3582 (2)	0.71844 (15)	0.02469 (14)	0.0712 (8)	
H8	0.4197	0.7036	−0.0257	0.085*	
C9	0.2220 (2)	0.68063 (15)	0.03839 (14)	0.0680 (8)	
H9	0.1743	0.6355	−0.0011	0.082*	
C10	0.5757 (3)	0.54733 (18)	0.27310 (18)	0.0803 (10)	
H10	0.6232	0.4895	0.2532	0.096*	
C11	0.4565 (3)	0.5510 (2)	0.32905 (16)	0.0944 (12)	
H11	0.4076	0.4961	0.3544	0.113*	
C12	0.4209 (3)	0.6485 (3)	0.34173 (13)	0.0959 (13)	
H12	0.3431	0.6724	0.3773	0.115*	
C13	0.5180 (3)	0.7050 (2)	0.29362 (17)	0.0829 (10)	
H13	0.5188	0.7747	0.2903	0.099*	
C14	0.6137 (2)	0.64251 (19)	0.25120 (15)	0.0731 (8)	
H14	0.6919	0.6616	0.2136	0.088*	

### Atomic displacement parameters (Å^2^)

**Table:**

	*U*^11^	*U*^22^	*U*^33^	*U*^12^	*U*^13^	*U*^23^
C1	0.0409 (11)	0.0494 (12)	0.0596 (12)	−0.0060 (8)	0.0131 (9)	0.0040 (9)
C15	0.0433 (10)	0.0462 (10)	0.0513 (11)	0.0066 (9)	0.0127 (9)	0.0041 (8)
C16	0.0615 (14)	0.0656 (15)	0.0611 (14)	0.0170 (12)	0.0308 (12)	0.0123 (12)
C17	0.099 (2)	0.0582 (14)	0.0644 (15)	0.0300 (15)	0.0443 (14)	0.0062 (13)
C18	0.116 (2)	0.0389 (11)	0.0612 (15)	0.0142 (13)	0.0411 (16)	−0.0014 (10)
C19	0.0687 (14)	0.0346 (10)	0.0447 (11)	0.0026 (8)	0.0199 (10)	0.0011 (7)
N1	0.0355 (7)	0.0365 (7)	0.0364 (8)	0.0017 (6)	0.0066 (6)	0.0008 (6)
N2	0.0418 (8)	0.0357 (8)	0.0374 (8)	0.0065 (6)	0.0085 (6)	0.0022 (6)
Zr1	0.02958 (7)	0.03000 (7)	0.03142 (7)	0.00527 (6)	0.00094 (6)	−0.00138 (6)
C5	0.0433 (12)	0.0566 (14)	0.093 (2)	0.0242 (10)	0.0149 (11)	0.0296 (13)
C6	0.0713 (16)	0.0367 (11)	0.108 (3)	0.0212 (11)	0.0257 (16)	0.0057 (13)
C7	0.0670 (17)	0.0398 (11)	0.118 (3)	0.0089 (11)	0.0268 (17)	0.0228 (14)
C8	0.0676 (14)	0.0726 (16)	0.0735 (16)	0.0295 (14)	0.0231 (15)	0.0402 (14)
C9	0.0630 (15)	0.0719 (17)	0.0690 (17)	0.0214 (13)	−0.0124 (13)	0.0270 (14)
C10	0.0732 (19)	0.095 (2)	0.0723 (19)	0.0222 (17)	−0.0423 (17)	−0.0064 (17)
C11	0.104 (3)	0.127 (3)	0.0521 (15)	−0.015 (2)	−0.0394 (19)	0.020 (2)
C12	0.086 (2)	0.163 (4)	0.0385 (14)	0.002 (2)	−0.0050 (13)	−0.0307 (18)
C13	0.081 (2)	0.092 (2)	0.0749 (19)	−0.0029 (18)	−0.0192 (16)	−0.0435 (18)
C14	0.0516 (15)	0.101 (2)	0.0671 (16)	−0.0002 (13)	−0.0210 (12)	−0.0239 (15)

### Geometric parameters (Å, °)

**Table:**

C2A—C1	1.518 (3)	C19—N2	1.343 (3)
C2A—H2A1	0.9800	C19—H19	0.9500
C2A—H2A2	0.9800	N1—Zr1	1.8428 (16)
C2A—H2A3	0.9800	N2—Zr1	2.3517 (16)
C3A—C1	1.521 (3)	Zr1—C5	2.5318 (16)
C3A—H3A1	0.9800	Zr1—C11	2.534 (2)
C3A—H3A2	0.9800	Zr1—C9	2.5570 (17)
C3A—H3A3	0.9800	Zr1—C12	2.5719 (19)
C4A—C1	1.514 (3)	Zr1—C6	2.5857 (17)
C4A—H4A1	0.9800	Zr1—C10	2.590 (2)
C4A—H4A2	0.9800	Zr1—C8	2.6255 (17)
C4A—H4A3	0.9800	Zr1—C7	2.6428 (17)
C2B—C1	1.519 (3)	Zr1—C13	2.649 (2)
C2B—H2B1	0.9800	Zr1—C14	2.6602 (19)
C2B—H2B2	0.9800	C5—C6	1.3933
C2B—H2B3	0.9800	C5—C9	1.3933
C3B—C1	1.516 (3)	C5—H5	0.9500
C3B—H3B1	0.9800	C6—C7	1.3933
C3B—H3B2	0.9800	C6—H6	0.9500
C3B—H3B3	0.9800	C7—C8	1.3933
C4B—C1	1.519 (3)	C7—H7	0.9500
C4B—H4B1	0.9800	C8—C9	1.3933
C4B—H4B2	0.9800	C8—H8	0.9500
C4B—H4B3	0.9800	C9—H9	0.9500
C1—N1	1.434 (3)	C10—C11	1.3807
C15—N2	1.346 (3)	C10—C14	1.3807
C15—C16	1.369 (3)	C10—H10	0.9500
C15—H15	0.9500	C11—C12	1.3807
C16—C17	1.373 (3)	C11—H11	0.9500
C16—H16	0.9500	C12—C13	1.3807
C17—C18	1.374 (3)	C12—H12	0.9500
C17—H17	0.9500	C13—C14	1.3807
C18—C19	1.373 (3)	C13—H13	0.9500
C18—H18	0.9500	C14—H14	0.9500
			
C1—C2A—H2A1	109.5	C5—Zr1—C10	155.98 (8)
C1—C2A—H2A2	109.5	C9—Zr1—C10	161.08 (8)
H2A1—C2A—H2A2	109.5	C12—Zr1—C10	51.29 (7)
C1—C2A—H2A3	109.5	C6—Zr1—C10	126.29 (8)
H2A1—C2A—H2A3	109.5	N1—Zr1—C8	119.55 (7)
H2A2—C2A—H2A3	109.5	N2—Zr1—C8	77.87 (6)
C1—C3A—H3A1	109.5	C5—Zr1—C8	51.80 (6)
C1—C3A—H3A2	109.5	C11—Zr1—C8	154.14 (8)
H3A1—C3A—H3A2	109.5	C12—Zr1—C8	129.69 (9)
C1—C3A—H3A3	109.5	C6—Zr1—C8	51.26 (6)
H3A1—C3A—H3A3	109.5	C10—Zr1—C8	130.42 (8)
H3A2—C3A—H3A3	109.5	N1—Zr1—C7	138.37 (7)
C1—C4A—H4A1	109.5	N2—Zr1—C7	99.39 (6)
C1—C4A—H4A2	109.5	C5—Zr1—C7	51.60 (6)
H4A1—C4A—H4A2	109.5	C11—Zr1—C7	126.55 (9)
C1—C4A—H4A3	109.5	C9—Zr1—C7	51.36 (6)
H4A1—C4A—H4A3	109.5	C12—Zr1—C7	99.06 (10)
H4A2—C4A—H4A3	109.5	C10—Zr1—C7	115.87 (8)
C1—C2B—H2B1	109.5	N1—Zr1—C13	131.80 (8)
C1—C2B—H2B2	109.5	N2—Zr1—C13	113.45 (7)
H2B1—C2B—H2B2	109.5	C5—Zr1—C13	105.91 (8)
C1—C2B—H2B3	109.5	C11—Zr1—C13	51.00 (7)
H2B1—C2B—H2B3	109.5	C9—Zr1—C13	125.24 (8)
H2B2—C2B—H2B3	109.5	C6—Zr1—C13	76.06 (8)
C1—C3B—H3B1	109.5	C10—Zr1—C13	50.46 (7)
C1—C3B—H3B2	109.5	C8—Zr1—C13	104.14 (9)
H3B1—C3B—H3B2	109.5	C7—Zr1—C13	75.64 (9)
C1—C3B—H3B3	109.5	N1—Zr1—C14	134.81 (8)
H3B1—C3B—H3B3	109.5	N2—Zr1—C14	83.90 (7)
H3B2—C3B—H3B3	109.5	C5—Zr1—C14	130.47 (7)
C1—C4B—H4B1	109.5	C11—Zr1—C14	50.87 (7)
C1—C4B—H4B2	109.5	C9—Zr1—C14	134.89 (8)
H4B1—C4B—H4B2	109.5	C12—Zr1—C14	50.52 (7)
C1—C4B—H4B3	109.5	C6—Zr1—C14	98.89 (7)
H4B1—C4B—H4B3	109.5	C8—Zr1—C14	104.58 (8)
H4B2—C4B—H4B3	109.5	C7—Zr1—C14	85.50 (8)
N1—C1—C4A	110.4 (2)	C6—C5—C9	108.0
N1—C1—C3B	113.7 (4)	C6—C5—Zr1	76.34 (7)
C4A—C1—C3B	135.8 (5)	C9—C5—Zr1	75.11 (6)
N1—C1—C2A	109.7 (2)	C6—C5—H5	126.0
C4A—C1—C2A	112.3 (3)	C9—C5—H5	126.0
C3B—C1—C2A	54.9 (4)	Zr1—C5—H5	114.8
N1—C1—C2B	109.9 (5)	C7—C6—C5	108.0
C4A—C1—C2B	58.6 (5)	C7—C6—Zr1	76.82 (6)
C3B—C1—C2B	107.6 (6)	C5—C6—Zr1	72.08 (7)
C2A—C1—C2B	57.3 (5)	C7—C6—H6	126.0
N1—C1—C4B	112.5 (4)	C5—C6—H6	126.0
C4A—C1—C4B	48.5 (4)	Zr1—C6—H6	117.1
C3B—C1—C4B	108.7 (6)	C8—C7—C6	108.0
C2A—C1—C4B	137.7 (5)	C8—C7—Zr1	73.98 (6)
C2B—C1—C4B	103.9 (6)	C6—C7—Zr1	72.29 (6)
N1—C1—C3A	108.0 (3)	C8—C7—H7	126.0
C4A—C1—C3A	109.9 (3)	C6—C7—H7	126.0
C3B—C1—C3A	52.6 (4)	Zr1—C7—H7	119.6
C2A—C1—C3A	106.4 (3)	C7—C8—C9	108.0
C2B—C1—C3A	142.0 (5)	C7—C8—Zr1	75.35 (7)
C4B—C1—C3A	63.3 (4)	C9—C8—Zr1	71.71 (6)
N2—C15—C16	123.0 (2)	C7—C8—H8	126.0
N2—C15—H15	118.5	C9—C8—H8	126.0
C16—C15—H15	118.5	Zr1—C8—H8	118.8
C15—C16—C17	119.4 (2)	C8—C9—C5	108.0
C15—C16—H16	120.3	C8—C9—Zr1	77.14 (7)
C17—C16—H16	120.3	C5—C9—Zr1	73.11 (7)
C16—C17—C18	118.2 (2)	C8—C9—H9	126.0
C16—C17—H17	120.9	C5—C9—H9	126.0
C18—C17—H17	120.9	Zr1—C9—H9	115.9
C19—C18—C17	119.9 (2)	C11—C10—C14	108.0
C19—C18—H18	120.0	C11—C10—Zr1	72.15 (8)
C17—C18—H18	120.0	C14—C10—Zr1	77.58 (9)
N2—C19—C18	122.2 (2)	C11—C10—H10	126.0
N2—C19—H19	118.9	C14—C10—H10	126.0
C18—C19—H19	118.9	Zr1—C10—H10	116.3
C1—N1—Zr1	168.93 (13)	C10—C11—C12	108.0
C19—N2—C15	117.30 (17)	C12—C11—Zr1	75.82 (9)
C19—N2—Zr1	118.78 (14)	C10—C11—H11	126.0
C15—N2—Zr1	123.60 (14)	C12—C11—H11	126.0
N1—Zr1—N2	95.64 (6)	Zr1—C11—H11	113.9
N1—Zr1—C5	87.67 (7)	C13—C12—C11	108.0
N2—Zr1—C5	122.03 (7)	C13—C12—Zr1	77.79 (8)
N1—Zr1—C11	86.16 (8)	C11—C12—Zr1	72.82 (9)
N2—Zr1—C11	103.93 (9)	C13—C12—H12	126.0
C5—Zr1—C11	134.01 (9)	C11—C12—H12	126.0
N1—Zr1—C9	90.23 (7)	Zr1—C12—H12	115.5
N2—Zr1—C9	90.26 (6)	C12—C13—C14	108.0
N1—Zr1—C12	101.27 (8)	C12—C13—Zr1	71.59 (8)
N2—Zr1—C12	128.65 (8)	C14—C13—Zr1	75.37 (7)
C5—Zr1—C12	106.90 (9)	C12—C13—H13	126.0
C9—Zr1—C12	137.15 (9)	C14—C13—H13	126.0
N1—Zr1—C6	115.46 (7)	Zr1—C13—H13	118.9
N2—Zr1—C6	128.29 (6)	C13—C14—C10	108.0
C11—Zr1—C6	117.67 (9)	C13—C14—Zr1	74.49 (8)
C9—Zr1—C6	52.00 (6)	C10—C14—Zr1	71.97 (8)
C12—Zr1—C6	86.33 (9)	C13—C14—H14	126.0
N1—Zr1—C10	105.18 (8)	C10—C14—H14	126.0
N2—Zr1—C10	77.57 (8)	Zr1—C14—H14	119.4
